# Becoming Housed During Palliative Care Enrollment: Identifying Drivers, Deterrents, and Directions for Future Care

**DOI:** 10.3390/ijerph21121596

**Published:** 2024-11-30

**Authors:** Ian M. Johnson, Rachel Doran, Nora Sullivan, Michael Enich, Michael A. Light

**Affiliations:** 1 Department of Social Work, University of Texas at San Antonio, San Antonio, TX 78249, USA; rachel.boal@my.utsa.edu; 2 School of Social Work, Rutgers University, Piscataway, NJ 08854, USA; nora.sullivan@rutgers.edu; 3 Department of Internal Medicine, University of Washington, Seattle, WA 98195, USA; menich2@uw.edu; 4 Palliative Care Training Center, University of Washington, Seattle, WA 98195, USA; lightma@uw.edu

**Keywords:** homelessness, housing, palliative care, multiple chronic conditions, end of life, healthy places

## Abstract

Homelessness is associated with accelerated disease progression, and housing placements are less likely when experiencing serious illness. Little research to date has focused on how to successfully secure housing placement during serious illness and end of life. This study aimed to address this gap by examining factors influencing housing placement among seriously ill palliative care patients experiencing homelessness. By conducting reflexive thematic analysis of medical record data of palliative care patients who became housed during enrollment (n = 16), three themes were identified: (1) trends in placement timing showing most accessed housing within seven months of palliative care involvement due to the relationship between homelessness, disease progression, and goals of care; (2) social support networks that were involved in attaining housing to varying degrees; and (3) changes in internal motivation, such as identity affirmation, relational tasks, and accepting limitation, driven by illness and dying processes. Findings underscore the need for integrated medical and social support, expanded housing options for the seriously ill, and adaptable psychosocial–spiritual care within the housing care continuum.

## 1. Introduction

Housing is often cited as a crucial health-related social need, most significantly as it impacts people experiencing homelessness (PEH). Homelessness can include living in an emergency shelter or public space not intended for habitation, a transitional space without a link to long-term housing, a highly supervised setting without an address for discharge, or experiencing an imminent threat of displacement from eviction, foreclosure, condemnation, or violence [1]. Multimorbidity—having more than one concurrent chronic health condition—is recorded among most Medicaid recipients with homelessness documented in their medical chart [2,3]. Chronic illness progression is accelerated and accentuated among people experiencing homelessness (PEH) [4,5], resulting in worse outcomes for chronic conditions like HIV/AIDS [6], cardiovascular disease [7], and mental illness [8]. Documentation of any housing-related social needs has also been associated with significant mental, behavioral, and neurologic disorders that cause long hospital stays [8]. Compounding health disparities include elevated risks of traumatic injury and infectious disease [9]. Because of such factors, PEH have a mortality rate four to nine times higher than their housed peers of comparable age, with an average life expectancy of 48–52 years [10].

For PEH in good health, establishing housing is often facilitated by two factors: (1) material stability, or the confidence that one’s basic needs can be regularly met [11,12], and (2) social support, or the instrumental and interpersonal help offered by people in one’s life [13,14]. For those who may need ongoing support for serious mental illness, substance abuse disorders, and/or biopsychosocial complexities, supportive housing is a well-documented pathway for becoming housed [15,16,17] and for reducing returns to homelessness [18]. However, high demand and low inventory mean that the threshold for entering supportive housing is increasing [19,20]. Protocols designed to determine the priority of housing-related needs based on an assessment of vulnerability (e.g., VI-SPDAT) have used functional impairment as a part of triage [21] but have shown racial bias [22,23], insufficiencies in recording domestic violence [24], and underassessment of medical conditions [25]. Existing literature indicates that serious illness, such as cancer and end-stage organ disease, is a risk factor for remaining homeless and notes the need for trajectory-based studies on how the severity of illness affects the multidimensional outcomes of PEH within housing programs [12].

Advanced stages of chronic illness or the emergence of serious illnesses may prompt a need for palliative care. Palliative care is specialized medical care for people living with a serious illness focused on relieving the symptoms and stress of illness and improving quality of life [26]. Palliative care is appropriate at any age and at any stage of a serious illness, and it can be provided along with curative treatment [27]. Home is most frequently identified as a preferred site of palliative care by both patients and caregivers [28]. Like their housed peers, older PEH have reported that secure housing influences their perceptions of a good death [29]. Despite these desires, when those with serious illnesses transition from disease-focused to comfort-focused care, it often requires a transition of caregivers, a physical site of care, or both [30]. These transitions can incite “biographical disruption” as early as diagnosis, resulting in changes in self-perception, daily routines, personal values, and future goals [31]. This process also shifts one’s social network, decreasing, turning over, or strengthening close ties [32]. Experiencing homelessness also exacerbates biographical disruption and erodes social networks [33], at the same time as it deleteriously impacts health [34]. Korkmaz-Yaylagul and Bas (2019) emphasized how social exclusion occurs when housing status, age, and disability intersect: a kaleidoscope of loss that includes place, mobility, social networks, resources, services, civic rights, and sense of utility [35]. Such measures of social connectedness are increasingly linked to health outcomes [36].

Research has responded to an increased international need for specialty care for people simultaneously experiencing homelessness and serious illness. The two known reviews of interventions and models of care for this population note that existing best practices focus on workforce development and the integration of specialty psychosocial–spiritual care, nursing, or advance care planning into shelters, hostels, and other temporary accommodations [37,38]. Palliative care and supportive housing systems have been recently urged to collaborate to preserve residence during serious illness [39], but efforts to do so have not yet been widely accomplished. Furthermore, no known research has explored housing placement during serious illness and end of life.

Identifying the facilitators and barriers to housing placements faced by seriously ill people is key to developing equitable systems of care. This study examines chart documentation from a subset of patients enrolled in a specialty palliative care outreach service who entered housing during their disease progression to contextualize the needs of patients experiencing homelessness. We ask the following questions: (a) What are the documented drivers of sustained housing placement among palliative care patients experiencing homelessness? and (b) what factors influenced the timing of housing placement among those simultaneously experiencing homelessness and serious illness?

## 2. Materials and Methods

### 2.1. Context

The research question guiding this study inductively emerged from a larger research study—Research, Action and Supportive Care at Later-Life for Unhoused People (RASCAL-UP). The RASCAL-UP study aimed to (1) characterize the disparities between desired and actual places of care experienced by unhoused older adults facing serious illness and (2) illustrate how healthcare and housing systems respond to the needs and wishes of people facing housing precarity and homelessness at the end of life. RASCAL-UP was conducted in partnership with a homeless palliative care team (HPC)—a specialty mobile outreach team composed of a social worker (MSW), a registered nurse (RN), and a nurse practitioner (ARNP) who offer medical and psychosocial support for people experiencing homelessness. The team operates in a large urban area in the Northwest U.S. Medical eligibility for the program includes a diagnosis of serious illness or advanced-stage chronic illness. Due to the mobile nature of HPC services, chart documentation provides insights into care in multiple locations at various moments in the progression of one’s illness. The specific research question for this study developed from previous findings identifying a subgroup of unhoused palliative care patients who received housing during palliative care enrollment and late into disease progression [40].

### 2.2. Collection

For the overall RASCAL-UP study, the research team was provided with a sample of 75 de-identified HPC patient charts. Data were collected from July to November 2021, when no human subjects data collection was permitted by the researchers’ home institution. Because of resource constraints, a purposive sampling strategy was deployed. Researchers chose to analyze the active rosters of the HPC team from March 2019, 2020, and 2021 to be able to monitor the potential effects of COVID-19 and seasonal variation. Institutional IRB did not require patient consent due to their de-identification prior to receipt; furthermore, the sample was largely deceased at the time of retrieval. For this study, 16 of the original 75 charts were included. The inclusion criterion was if the patient had obtained housing within their enrollment in palliative care. The charts excluded from this analysis had other trajectories of care outlined by Johnson and Light (2023) [40].

### 2.3. Analysis

Descriptive statistics were generated in SPSS Version 27 [41]. Before engaging in the coding process, the first author generated narrative summaries of each case, meant to provide a supplemental “birds-eye” perspective when compared to individual codes [42]. Reflexive thematic analysis [43] was then employed to analyze the text of chart documentation. The first author performed systematic coding on original chart documentation using Dedoose [44]. In the second round of coding, the first and last authors reviewed memos, initial codes, and narrative summaries to synthesize codes and develop themes. The research question was inductively developed during this process. The second, third, and fourth authors reviewed narrative summaries and chart data of the subsample, sharing their initial codes and memos. The full research team then engaged in a dialogue to compare, reconcile, and finalize themes.

### 2.4. Sample

The average age of the sample was 58 with a median of 61 and a range from 30 to 72. The sample was predominantly White (56.3%) and male (75%). About 37.5% were referred to palliative care for cancer, while the rest of the sample was referred for late-stage chronic illnesses such as chronic obstructive pulmonary disease (COPD), end-stage renal disease (ESRD), or congestive heart failure (CHF). The mean duration of enrollment in palliative care services was 22.5 months. Participant details are illustrated in Table 1; in summarizing patient trajectories, hospitalizations were included when they resulted in a subsequent change of sleeping place.

## 3. Results

Our analysis elucidated three primary themes: (1) trends in placement timing, indicating that the majority of the sample accessed housing within seven months of palliative care involvement due to the relationship between homelessness, disease progression, and goals of care; (2) social networks’ involvement in sustained housing attainment, which helped patients to varying degrees; and (3) shifts in internal motivation for housing driven by developmental tasks relevant during illness and dying processes, such as identity affirmation, relational tasks, and accepting limitation.

### 3.1. Trends in Placement Timing

**Figure 1:**
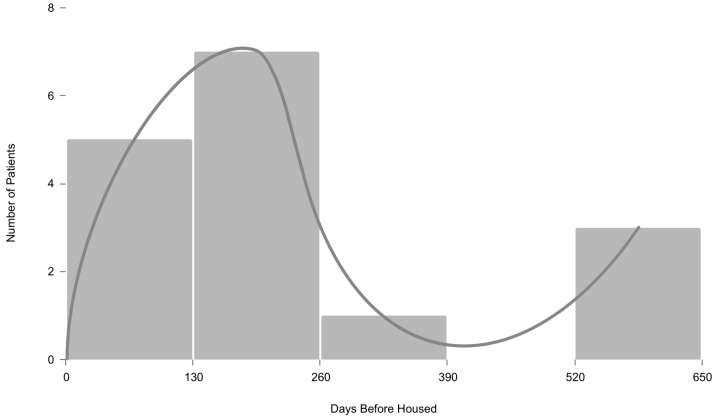
Histogram depicting days before accessing housing across sample (n = 16).

The tension between existing barriers to housing and disease progression influenced housing placement. While 12 patients within the sample were housed within seven months of palliative care enrollment, 4 experienced further delays in housing placement. Figure 1 depicts the distribution of patients across the timeline in which they secured housing. The likelihood of securing housing first increased as the patient received a serious illness diagnosis and became connected with formal support. While barriers to housing were still present, new access to psychosocial and medical services expedited housing placement. For four patients, however, homelessness persisted after seven months of enrollment in care with common barriers to service access associated with homelessness such as documentation, service disconnects, and behavioral health becoming more pronounced when experiencing serious illness. This had the effect of driving these patients away from services despite a greater need for formal support prompting significant delays in securing stable housing.

#### 3.1.1. Palliative Care Involvement Increases Access to Housing

Once diagnosed with serious illness, patients are linked to increased resources, driven by both formal avenues for housing and a collective desire to house the patient. Palliative care’s involvement in housing increased healthcare access in tandem with housing, reconnected services despite system failure, and provided resources to maintain housing.

Despite initial resistance to starting treatment, Kevin welcomed care from the hospice team after regaining mobility after surgery (MSW, 2 April 2019). Shortly thereafter, Kevin’s palliative care team facilitated access to housing services and connected him with formal support to provide food and income (MSW, 3 April 2019; 4 April 2019; 17 April 2019; 15 May 2019), which also facilitated greater success in accessing medical appointments. Once stabilized in his apartment with wrap-around end-of-life services, the palliative care team discharged him. Before discharge, Kevin expressed his sentiment regarding these outcomes saying, “I am really happy” (RN, 25 June 2019).

While hospitalized, the HPC team advocated for Kyle to access a respite bed at an emergency shelter despite communication difficulties between healthcare and shelter services during discharge and lack of bed availability (MSW, 9 April 2020). Higher acuity care from the palliative care team ensured that patient needs were met where other institutional supports failed. Kyle’s discharge to the respite bed reduced reported feelings of being “stuck” (MSW, 9 April 2020) and aligned with his goals of navigating end of life in the community and beyond hospital walls.

Similarly, Leo received access to notary services and housing application assistance (RN, 22 August 2017) within four months of being referred to palliative care by his housing case manager (RN, 18 April 2017). Shortly after, Leo acquired a new wheelchair (RN, 20 November 2017) before securing a unit in permanent supportive housing along with his wife (MSW, 5 April 20118). The palliative care team later supported Leo’s wife in navigating ways to maintain housing in the event of Leo’s death (MSW, 5 April 2018).

#### 3.1.2. Existing Barriers and Serious Illness Complexity Delay Housing Access

In order to secure housing, patients must overcome barriers related to documentation, service disconnect, varying service reactions to substance use as pain management, and sustained trauma. Although serious illness diagnosis facilitated connections to the palliative care team, it did not eliminate existing barriers to housing and created new barriers related to disease trajectory. When these barriers are compounded by limited and ineffective housing systems, patients experience longer delays and shorter periods of sustained housing.

Accessing documentation was a key step in gaining housing across the sample. Natalie was unable to move into housing until she secured her social security card (RN, 8 April 2019), which delayed her ability to move out of a shelter that she felt threatened her physical and psychological well-being. Natalie noted the shelter limited access to her oxygen concentrator, provided poor environmental conditions that exacerbated her COPD symptoms, and exposed her to violence (MSW, 6 March 2019).

The interpretations of substance use and consequences for using vary by service. For instance, Gina, a patient without a substance abuse history, was “kicked out of [shelter] for having medical THC and opioids for her cancer pain, since the shelter is substance-free” and her social worker noted “HPC did not realize that Rx opiates would not be allowed. Patient had to return to [medical respite facility]” (MSW, 10 September 2020).Multiple changes in locations also limited her ability to establish long-term housing case management services. This incident was a breach of trust with housing case management staff that created service disengagement (MSW, 23 February 2021). Documentation from other patient charts also illustrated how timely and appropriate housing placement was predicated on trust. In early palliative care treatment, Amy, a patient with opioid use disorder, made an “unprompted assertion” that she was not using illicit substances (ARNP, 9 November 2018); however, once housed, she informed team members that she had used illicit substances throughout treatment to assist in pain management (RN, 27 November 2019). Trust in the care relationship is essential for timely and appropriate housing referral. Inconsistent philosophies of acceptable pain management across services distance seriously ill patients from accessing housing.

Trauma was a common experience among patients in the sample, which reframed the way patients navigated other aspects of their serious illness. While engaged in care, Gary lost four family members—his nephew, sister, daughter, and brother-in-law (RN, 11 January 2016; 26 September 2016; 1 February 2019; 1 October 2019). Grief may have affected Gary’s ability or motivation to share his internal experiences with care professionals, as noted in an RN note (24 June 2019) where Gary reported “he feels at baseline and functions at baseline”, but by observation “…appears unhealthy” and “exhibits a depressed mood” (RN, 24 June 2019). Kyle endorsed suicidal ideation upon discharge from the hospital, when he discovered he no longer had a bed at the shelter or access to housing case management services there (MSW, 9 April 2020). HPC staff assisted him in securing housing and documented the need for trauma-informed care in Kyle’s charts: “Consider trauma history in care interactions and employ de-escalation skills as standard of practice” (RN, 22 February 2019). Similar recommendations are documented for interactions with patients Gina (MSW, 10 September 2020), Ivan (RN, 6 March 2018), and Leo (MSW, 23 January 2019); yet the need for trauma-informed mental health intervention to lower barriers to housing access persisted across this patient population.

Accessing housing at one point in time does not guarantee that housing will continue. Home and community-based services faced limitations in scope and collaboration, which consequently created precarity for community-dwelling clients. For instance, Jesse missed his ride for his appointment due to the shelter staff not scheduling it (RN, 26 March 2020). Once Jesse secured public housing, he was stuck in his house lying on the floor for three days before managing to get himself to the hospital (RN, 11 March 2021). When Jesse recovered and hoped to return to his apartment, the palliative care team contacted his case manager, but she was unsure of the status of his apartment and his belongings (RN, 21 April 2021). Disconnection between transportation, medical, and housing services limited their capacity to meet Jesse’s care needs and hindered Jesse’s capacity to reaccess housing.

### 3.2. Social Networks of Care

Social networks facilitated access to housing through assistance with care continuity, documented through providing physical space, watching over patient whereabouts and well-being, and mediating cultural barriers. Data also suggested that social networks helped preserve housing after placement in ways that may be unique to those with serious health conditions through instrumental, material, and emotional support. However, there were also documented ways in which social networks stymied placement in housing, such as uncertainty about the plausibility of patient goals of care, care partner emotional avoidance, and social losses associated with relocation.

#### 3.2.1. Social Networks Facilitating Housing Placement

Medical acuity and severity can exacerbate fluctuations in patients’ sleeping locations, but collaboration between social networks and formal care professionals can offset ruptures in care that might affect housing placement. The day prior to his enrollment in palliative care, patient Ivan and his boyfriend were evicted from a motel they lived in for nearly five months. Ivan’s primary concern when meeting the HPC nurse practitioner in the hospital was finding a place to live, expressing that he could not “make it on the streets” given his chronic wounds and difficulty ambulating independently (ARNP, 7 January 2017). For the next four months, Ivan moved between the hospital and medical respite and stayed with his boyfriend in various motels while his healthcare team identified pathways to secure housing. Ivan’s transience through these settings contributed to a lapse in communication with formal professional assistance until Ivan’s boyfriend secured his own supportive housing studio. While housing rules restricted Ivan to limited amounts of time staying with his partner, it helped him reconnect with care after this period of disengagement. His goal of obtaining housing persisted as noted in an RN visit (8 August 2017) where Ivan shared, “sometimes I have to sleep on the streets and at any time they could boot me out of here. I really need a place, man”. The HPC team collaborated with Ivan’s boyfriend’s housing case manager and Ivan obtained housing three months later (MSW, 27 December 2017).

Another example is Doug, who was in and out of motels and hospitals when his outpatient clinic referred him to palliative care (ARNP, 14 November 2018). Difficult to track down, the palliative care team was able to conduct an intake with Doug because his daughter’s friend worked at the front desk of another service site (RN, 7 January 2019). While the Veterans Administration was the primary source of housing and health support for Doug, his extended social network enabled placement into supportive housing by creating a safe and acceptable location for Doug to access services and a consistent contact for HPC to coordinate with (RN, 30 January 2019).

In the case of a patient, Hunyh, a friend helped reconcile the homeless service sector expectations with cultural norms and values. After over a year of assisting Hunyh in managing health crises while living unsheltered and obtaining the necessary documentation and paperwork to be eligible for supportive housing, the HPC RN spent another month (RN, 8 May 2019; 12 May 2019; 14 May 2019; 24 May 2019; 29 May 2019) attempting to help Hunyh successfully attend an interview for a housing unit. In documentation, Hunyh was reported to ascribe his refusal to his own poor hygiene (RN, 29 May 2019). Throughout his treatment, a friend of Huynh’s, also Vietnamese, was not only able to assist him with hygiene and personal upkeep but also communicated the cultural significance of hygiene and its relationship with Huynh’s personal sense of dignity (RN, 11 June 2019).

#### 3.2.2. Social Networks Sustaining Housing Placement

Social networks were also observed to help sustain housing in moments where health created precarity, primarily by supplementing formal services with assistance in activities of daily living. Patient charts documented that romantic partners and friends helped with cleaning, transportation and medical escort, and groceries. When insurance rejected Leo’s claim to get an electric scooter, something he believed he needed to comfortably leave his apartment, he and his wife were able to purchase a used one through informal connections (RN, 24 July 2019).

Support from informal care partners extended beyond the material and instrumental. For Gary, family members provided holistic support once he secured housing and sustained sobriety. His former wife encouraged him to seek treatment for liver disease (MSW, 14 November 2016). Gary’s brother financially sponsored domestic travel to visit family during holidays and other important family events (ARNP, 7 December 2015; RN, 27 November 2016; ARNP, 9 October 2019). Gary’s brother was among a web of supporters who encouraged Gary to remain sober, noting that they attended AA meetings during family trips (RN, 28 November 2016) and attended a 12-Step conference together (ARNP, 19 July 2016). When Gary was threatened with eviction due to the behavior of one of his guests, his son offered to house him for three weeks, which helped offset Gary’s anxiety about housing loss and mitigated potential conflicts with property management (ARNP, 21 March 2019; 17 April 2019).

#### 3.2.3. Social Networks Complicating Housing Access

While informal networks of care were often assets, they could also increase complications. For some patients, family members’ discomfort in engaging in conversations about death and dying impacted housing security. At the beginning of palliative care service engagement, Leo’s wife was crucial in escorting Leo to medical appointments, obtaining durable medical equipment, and applying for benefits (MSW, 5 April 2018). However, as Leo’s disease progressed, she became increasingly anxious and disengaged:

“His spouse was in the bathroom for the first part of visit but leaves when patient begins discussing his feelings regarding end of life… [patient] also says his spouse seems more depressed and does not want to accompany him for activities. Patient wants to spend time with her so he stays home. Patient notes lack of activity has been impacting the amount of time thinking about death and feelings of depression…says that spouse seems avoidant of conversations about his death.”

(MSW, 23 January 2019)

While it is natural and expected for care partners to engage in their own process of grief, Leo required his partners’ continued engagement and participation in formal health and housing case management services to stay in the community.

Some patients discussed family during care planning, but their actual capacity to assist with care was never determined. For example, Kyle’s serious illness goals focused on longevity so that he could have time to reconnect with his 6-year-old daughter (MSW, 17 March 2020). Early in treatment, Kyle’s health goals and acceptance of housing seemed contingent on the extent to which other family members were willing to provide care for him at home (MSW, 9 April 2020). Similarly, Carlos only wished to obtain housing in his birth country of Mexico and believed his brother would be willing and able to donate a kidney (MSW, 19 May 2019). Neither family-provided care nor kidney transplant were practical or clear options, and intermittent contact with his mother and brother created health and housing impasses, as Carlos declined to consider alternative options (MSW, 19 June 2019).

Both Peter’s and William’s chart notes indicate that the social networks created while living in a men’s shelter made transitioning into housing more challenging. Both men resisted housing during treatment due to concern over losing social support. For Peter, a small network of close friends at the shelter provided cellular phone access (RN, 21 January 2021) and instrumental support like assistance with medication management and ambulation (RN, 27 April 2020). After obtaining an apartment, Peter expressed to the HPC team, “you can come visit when I go home—it gets lonely there” (RN, 16 October 2020). After roughly five months spent between hospitals and medical respite, William obtained a housing voucher and was discharged to an emergency shelter until he could find housing. Once back at the shelter, he lost momentum in his search for an apartment, telling the HPC social worker, “I’m really well liked down here” (10 March 2020). William was rehospitalized due to COVID-19 and pneumonia shortly thereafter and then was referred to temporary accommodations in a hotel during citywide efforts to reduce the spread of infection. Now isolated from his shelter community, William used his housing voucher to secure an apartment.

### 3.3. Internal Motivation Shifts Toward Housing

During their serious illness trajectories, patients were documented by palliative care professionals to share internal motivators for accessing housing. While patients likely had existing aspirations for exiting homelessness prior to serious illness, serious illness diagnoses and limited prognoses appeared to shift patient priorities and accelerate an urgency for housing access. Developmental tasks such as affirming one’s own identity as a survivor, reprioritizing an environment to tend to important relationships, and reconciling with one’s limitations were all identified in the process of obtaining housing during palliative care.

#### 3.3.1. Housing as Identity Affirmation

For some patients, housing took on symbolic meaning as a manifestation of survivorship. For these patients, obtaining adequate, stable housing meant they had endured and overcome concurrent and intersecting health and housing barriers. The attitude of survivorship not only created momentum toward securing housing but also contributed to a sense of meaning-making once housing was obtained.

Natalie found that attaining housing empowered her to connect to a positive self-image. Natalie resided in an emergency shelter for the first three months she was enrolled in palliative care. During that time, she was amenable to nursing home placement and positively reflected on her time living in a tent with her cat, clearly asserting that the emergency shelter was last on her list of preferred sleeping places. When her housing application was accepted, the palliative care team documented as follows:

“She is so grateful for the opportunity that she begins to cry. She talks at length about the abuses and inhumane treatment she has experienced at [shelter] from both fellow stayers and staff. She reports that she is saving up for a generator and is planning on moving into a tent she has already bought on March 28 if housing doesn’t come through: ‘A person can only take so much, and I’ve reached my limit. I’m going crazy in here. How long are you supposed to take it without fighting back or leaving?’ … She speaks about trauma and its effect on her. She is concerned she has been permanently changed through her stay.”

(MSW and RN, 8 April 2019)

Nine months after obtaining housing, now entwined in a protracted legal battle with the shelter, Natalie shared with the HPC social worker her calling is to “stand up for other people who can’t do it for themselves” (19 January 2020). Away from the source of trauma and in her own apartment with an on-site nurse, Natalie declined further assistance from the palliative care team, choosing to invest her time in seeking justice.

Quinn, living with heart failure, slept outdoors without a tent at the beginning of her palliative care involvement. Documentation from Quinn’s chart illustrated how she began making a proactive choice to stay connected with services, selecting a sleeping place closer to the hospital as her health declined and winter weather set in (ARNP, 21 November 2018). When her application for a permanent supportive housing studio was approved, she was documented as saying: “I have a place, I just have to survive for ten more days” (ARNP, 18 January 2019). Once housed and connected to visiting mental health treatment, Quinn did not require much from palliative care services, staying in her studio until her death 18 months later.

Despite a terminal breast cancer diagnosis, the moment Gina secured housing signified victory over the fate of dying while unhoused—a moment of triumph not only for her but over a history of family deaths caused by breast cancer (MSW, 17 March 2020). She was documented to tell the palliative care team, “I made it. I survived” (MSW, 3 August 2021). During the first palliative care team visit to her new apartment, Gina was documented as saying: “I have everything now. I showed cancer. I showed [city]. I did this for my mother and all the women in my family. I’ve got this” (MSW, 5 August 2021). 

#### 3.3.2. Housing as a Place for Connection

For some patients, serious illness accelerated the need for home space that could facilitate relationship growth, healing, and connection. Ivan discussed willing his vehicle and Social Security back pay to his boyfriend after becoming housed, reported saying, “I want him to be happy and remember me” (RN, 19 March 2019). Shortly after moving in, Ivan discussed with the palliative care nurse the significance of celebrating Christmas and baking a cake together as a gift of memory-making (RN, 27 December 2017). 

William lived in an emergency shelter for several years, and among his limited possessions were two stuffed animals that served as symbolic representations of his two children (RN, 7 January 2020). Throughout treatment, he made clear a psychosocial goal of reconnecting with his estranged adult children (MSW, 19 October 2019). Obtaining housing was a facilitator of this goal for William—in the five months of living in a studio apartment before his final hospitalization and death, William was able to reconnect with family after 20 years apart (MSW, 21 September 2020).

Living with a progressive serious illness prompted Leo to identify stable housing as a legacy goal. Once housed, Leo communicated increased distress regarding end of life, stating, “I have more to live for than ever” (MSW, 23 January 2019). In Leo’s shift towards longevity as a treatment goal once housed, he also expressed to the HPC social worker that he wanted to ensure after his death that his wife would be able to keep their housing and receive his SSDI payments.

#### 3.3.3. Housing as Accepting Limitation

When faced with the realities of serious illness, disease progression, and limited prognosis, patients were sometimes motivated to confront and reconcile ambivalence regarding personal and health goals. As Carlos’ condition progressed and he inched closer to permanent supportive housing placement, he stopped planning visits to Mexico (MSW, 15 May 2019) and communicated a loss of interest in pursuing curative treatments (MSW, 4 June 2019). Ryan was a former fisherman whose heart attack while living in a tent encampment prompted a palliative care referral. Ryan’s health needs and life goals were in conflict early in treatment, as he wanted to return to Alaska for a final season of work leading him to leave medical respite care and disengage from case management services that had been assisting him toward housing. Ryan was not able to make it to Alaska, experienced a return to substance use, and returned “full of shame and feeling so sorry” (RN, 24 July 2019) that he disengaged from all services for 14 months. A hospitalization and diagnosis of T-cell lymphoma prompted re-engagement with care and drove Ryan’s reprioritization of housing over other psychosocial goals.

## 4. Discussion

Housing is both a symptom management and psychosocial–spiritual intervention for people experiencing homelessness and living with serious illness. This study introduced exploratory ways in which specialized palliative care involvement could assist in obtaining housing and overcoming barriers that caused delays to housing placement. Mobile health services can enhance chronic health management and address social determinants of health. In a review of U.S. mobile health clinics, Yu et al. (2017) identified intervention efficacy in assessing urgency, providing convenience, earning trust, increasing screening and preventative care, and improvements in self-reported ability to manage chronic illness [45]. Over the past two decades, palliative care services for people experiencing homelessness have been explored globally—for example, the uptake and cost of shelter-based palliative care [46], completion of advance care plans at homeless shelters [47,48], community-based harm reduction services as an entry point to serious illness care [49], mobile outreach [50], and specialty social model hospices [51,52,53]. Quality appraisal of this body of research indicates a need for improved intervention and implementation outcome measurement [38]. Workforce development interventions aimed at supporting PEH with serious illness must be developed and tested in healthcare settings [54], shelters [55], and supportive housing [37].

This study’s findings reiterated previously established barriers to housing placement, such as documentation [56], disconnects between service milieus [57], abstinence-only recovery frameworks [12,58], and persistent trauma [59]. Such barriers are more complex and nuanced for people with life-limiting illnesses, given the developmental tasks of end of life [60], increased frequency of forced interactions with emergency medical professionals [61,62], and the influence of pain and other symptoms [63].

As demonstrated by the barriers outlined in the results, assertive engagement—a trauma-informed, harm-reduction approach to re-engaging clients in services despite previous difficult interactions [64]—mitigates both housing and healthcare hurdles for people concurrently facing homelessness and serious illness. Findings demonstrate the importance of developing and sustaining interprofessional dialogue and training on how best to address barriers to care and housing, in addition to Street Medicine [65] and health outreach interventions to increase healthcare and housing referrals.

Findings from this study illustrated how biological and chosen families alike moved housing placement forward by offering material, emotional, social, and instrumental assistance. Research indicates that homeless individuals who receive instrumental support from within their social networks are more likely to exit homelessness [11] and remain stably housed [14], and to experience improved health outcomes [66]. Conversely, when housing systems limit access to outside social support due to single occupancy policies or other restrictive regulations, one’s ability to manage serious illness and health crises may worsen [20]. Amid functional decline during serious illness, additional social support assists in navigating new health needs. Informal support can be particularly meaningful when past experiences of relational harm, such as manipulation or violence, disincentivize seeking help from formal services [67,68], or when professional quality of care is not available or sufficient [69]. Expanding financial incentives for informal carers and eligibility criteria for community-based care can help sustain engagement from the social networks of PEH facing serious illness [70]. Findings also indicated that in times of serious illness, some care partners of PEH enrolled in palliative care may benefit from enhanced support. It may therefore be of value to explore adaptations to serious illness communication, grief support, and death preparedness interventions with and for care partners.

Findings from the study also demonstrate how intrinsic factors influencing housing service engagement may shift considering patients’ awareness of their prognosis and their psychosocial priorities in later life. This trend was presented through patient expressions that housing was a symbolic representation of their identity, a place to facilitate relational end-of-life tasks, and a means to resolve ambivalence and tension around competing hopes and goals. A past analysis of older homeless women illustrated how maintaining a sense of self while homeless required both a sense of membership to the experiences of homelessness and aging while simultaneously resisting labels and associated meanings of “older” and “homeless” [71]. Work related to identity and selfhood is also asserted as central to transitioning between living and dying [72]. Obtaining housing during a psychosocial–spiritual juncture may facilitate a meaning-making process surrounding both one’s housing loss and serious illness.

End-of-life processes can also prompt family reunification [73] and deepen bonds with loved ones [74]. Housing placement is also well-documented as a transitional period for rebuilding social networks [33], including with estranged or strained family members [12,75]. Findings indicating that reprioritization of housing placement was linked to coming to terms with the acquiescence of other goals mirror literature on relocation among housed people amidst acquired disability [76], aging [30], and end-of-life care [77]. Such studies note that while distress is one outcome, relocation may also conjure feelings of resolution, surrender, or integration, leading to a reduction in holistic suffering for patients and care partners. A dynamic tension exists at the end of life, in which relocation may threaten one’s agency in their own identity, connectedness, and sense of place [78], a phenomenon amplified among those with lower income and mental health disorders [79]. Housing, community mental health, clergy, and healthcare practitioners should consider how to promote agency and positive self-concept in instances of relocation in later life through evidence-based practices like existential psychotherapies, life review, and dignity therapy.

While longitudinal healthcare documentation contains valuable information about patient care, its use in research poses challenges. The content of medical chart data differed greatly across the three documentarians; while in part due to role, such variation may indicate undecipherable value differences between what is worth recording to individual professionals and raised questions about data completeness. Resource constraints were a factor in the sampling approach and collection strategies. Rigorous future research aimed at supporting later-life exits from homelessness would include a wide range of data sources, including direct involvement from palliative care patients experiencing homelessness.

## 5. Conclusions

This study examined factors influencing successful and timely housing placement for palliative care patients experiencing homelessness. Findings revealed that palliative care professionals played a pivotal role in housing placement, often in the face of barriers such as lack of documentation and service fragmentation, which delayed access. Strong social networks facilitated housing transitions and prevented a return to homelessness, but their impact varied widely. As patients’ health declined, their housing needs evolved, emphasizing the importance of identity affirmation, relationship building, and acceptance of illness. These findings indicate a need for targeted approaches to eliminating barriers to housing placement during serious illness, workforce development that helps integrate homelessness response and palliative care services, and harm reduction-focused, trauma-informed psychosocial–spiritual care that makes space for the complexities of concurrent homelessness and serious illness.

## Figures and Tables

**Table 1:** Sample details.

Pseudonym	Demographic Details	Months Enrolled in Palliative Care	Trajectory of Care
Amy	A 62-year-old Black woman referred for chronic obstructive pulmonary disease	27.1	Hospital Skilled nursing Doubled up Supportive housing Hospital
Carlos	A 53-year-old Latino man referred for end-stage renal disease	5.7	Hospital Tiny home village Hospital Supportive housing
Gary	A 60-year-old Black man referred for liver cancer	51.1	Hospital Tiny home village Hospital Supportive housing
Gina	A 51-year-old Black woman referred for breast cancer and pulmonary embolism	18.0	Crisis diversion center Hospital Respite Shelter A Hospital Respite Shelter B Short-term rental Respite Low-income housing
Ivan	A 58-year-old White man referred for congestive heart failure and chronic kidney disease	26.4	Hotel Hospital Respite Doubled up Shelter Hospital Respite Doubled up Supportive housing Hospital
Kevin	A 63-year-old White man referred for prostate cancer	9.6	Respite Hospital Temporary housing Supportive housing
Kyle	A 30-year-old Black man referred for congestive heart failure and pulmonary embolism	4.7	Respite Shelter Hospital Shelter Hospital Supportive housing
Leo	A 62-year-old White man referred for colorectal cancer	45.6	Vehicle Hospital Respite Motel Hospital Supportive housing Hospital
Hunyh	A 57-year-old Southeast Asian man referred for pulmonary embolism/stroke	45.8	Hospital Doubled up Hospital Vehicle A Hospital Street Hospital Doubled up Hospital Street Hospital Street Supportive housing
Natalie	A 63-year-old White woman referred for chronic obstructive pulmonary disease	10.6	Hospital Shelter Supportive housing
Quinn	A 54-year-old White woman referred for pulmonary embolism and chronic obstructive pulmonary disease	31.5	Hospital Respite Hospital Street Hospital Respite Supportive housing
Ryan	A 50-year-old White man referred for lymphoma and congestive heart failure	35.8	Street Hospital Respite Street Hospital Respite Street Supportive housing
Peter	A 68-year-old White man referred for chronic obstructive pulmonary disease	15.6	Shelter Hospital Hotel A Hotel B Low-income senior housing
William	A 67-year-old White man referred for esophageal cancer	12.4	Hospital Respite A Hospital Respite B Shelter Hotel Section 8 housing Hospital
Jesse	A 72-year-old Black man referred for congestive heart failure	16.7	Shelter Hospital Hotel A Hotel B Section 8 housing Hospital Skilled nursing
Doug	A 65-year-old Indigenous American man referred for leukemia and cirrhosis	3.7	Hospital Hotel Temporary housing Veteran supportive housing Skilled nursing

## Data Availability

Data are not publicly available due to privacy and ethical restrictions.
